# Syntenin-1-mediated small extracellular vesicles promotes cell growth, migration, and angiogenesis by increasing onco-miRNAs secretion in lung cancer cells

**DOI:** 10.1038/s41419-022-04594-2

**Published:** 2022-02-08

**Authors:** Okhwa Kim, Cheol Hwangbo, Phuong Thao Tran, Jeong-Hyung Lee

**Affiliations:** 1 Department of Biochemistry, College of Natural Sciences, Kangwon National University, Chuncheon, 24341 Republic of Korea; 2 Kangwon Institute of Inclusive Technology, Kangwon National University, Chuncheon, 24341 Republic of Korea; 3 Division of Applied Life Science (BK21 Four), Division of Life Science, College of Natural Sciences, Gyeongsang National University, Jinju, 52828 Republic of Korea

**Keywords:** Cancer microenvironment, Non-small-cell lung cancer, Oncogenesis

## Abstract

Small extracellular vesicles (sEVs) play a pivotal role in tumor progression by mediating intercellular communication in the tumor microenvironment (TME). Syntenin-1 induces malignant tumor progression in various types of human cancers, including human lung cancer and regulates biogenesis of sEVs. However, the function of syntenin-1-regulated sEVs and miRNAs in sEVs remains to be elucidated. In the present study, we aimed to demonstrate the role of oncogenic Ras/syntenin-1 axis in the release of sEVs and elucidate the function of syntenin-1-mediated miRNAs in sEVs in lung cancer progression. The results revealed that oncogenic Ras promoted the release of sEVs by inducing syntenin-1 expression; disruption of syntenin-1 expression impaired the release of sEVs as well as sEV-mediated cancer cell migration and angiogenesis. Moreover, we identified three miRNAs, namely miR-181a, miR-425-5p, and miR-494-3p, as onco-miRNAs loaded into syntenin-1-dependent sEVs. Remarkably, miR-494-3p was highly abundant in sEVs and its release was triggered by syntenin-1 expression and oncogenic Ras. Ectopic expression of the miR-494-3p mimic enhanced the migration and proliferation of lung cancer cells as well as tube formation in endothelial cells; however, the miR-494-3p inhibitor blocked sEV-mediated effects by targeting tyrosine-protein phosphatase nonreceptor type 12 (PTPN12), a tumor suppressor. sEVs promoted tumor growth and angiogenesis by downregulating PTPN12 expression; however, the miR-494-3p inhibitor significantly suppressed these effects in vivo, confirming that miR-494-3p acts as a major onco-miRNA loaded into lung cancer cell-derived sEVs. Eventually, the oncogenic Ras/syntenin-1 axis may induce cancer progression by increasing miR-494-3p loading into sEVs in lung cancer cells in the TME.

## Introduction

Extracellular vesicles (EVs) are nanosized membrane-bound vesicles that are released in a majority of cell types; these include (1) small extracellular vesicles (sEVs), known as exosomes, with a size range of ~40–160 nm, which are released upon fusion of multivesicular bodies (MVBs) with the plasma membrane, and (2) microvesicles (MVs), known as ectosomes with a size range of 100–1000 nm, which are generated by the direct outward budding of the plasma membrane [1, 2]. sEVs are generated during invagination of the endosomal limiting membrane and formation of intraluminal vesicles (ILVs), thereby resulting in specialized endosomal compartments, known as MVBs. Thereafter, the ILVs are released as sEVs by the fusion of MVBs with the plasma membrane and via exocytosis [2, 3]. The endosomal sorting complex required for transport (ESCRT)-dependent and ESCRT-independent pathways mediate the formation of ILVs and sorting of protein cargo [2, 4, 5].

Syntenin-1 is a multifunctional adapter protein comprising a tandem PDZ (PSD-95/Dlg/ZO-1) domain that regulates numerous cancer cell functions, such as proliferation, migration, invasion, EMT, metastasis, and angiogenesis [6–8]. Syntenin-1-syndecan-ALIX (ALG-2-Interacting Protein X) is required for the biogenesis of sEVs [9]. Syntenin-1 directly interacts with ALIX through LYPX(n)L motif, and ALIX further connects the syntenin-1-syndecan complexes to ESCRT components that are crucial in ILV formation [9, 10]. This process is regulated by the small GTPase ADP ribosylation factor 6 (ARF6) and phospholipase D2 [10]. Moreover, syntenin mediates tyrosine kinase Src function in sEV-mediated cell-to-cell communication by controlling exosome activity and biogenesis [11]. Src stimulates the secretion of sEVs by phosphorylating syntenin Y46 and the DEGSY motif of the syndecan cytosolic domain [11]; however, tyrosine phosphatase Shp2 decreases the secretion of sEVs by dephosphorylating syntenin Y46 [12].

The sustained growth, invasion, and metastasis of cancer cells depend on intercellular communication in the tumor microenvironment (TME). sEVs crucially mediate the intercellular communication between cancer cells and other cells in the TME [13]. Increasing evidence suggests that cancer cells secrete higher amounts of sEVs than normal cells. In particular, certain cancer cells overexpress ESCRT proteins and syntenin-1 [14, 15]. Furthermore, activation of oncogenic signaling pathways stimulates sEV production; for example, epidermal growth factor receptor variant III (EGFRvIII) and K-RasV12 stimulate the release of sEVs [16, 17]. In addition to increased sEV release, the cancer cells release sEVs that differ in molecular cargo by loading oncogenic molecules, such as proteins and miRNAs [13, 18–20]. Oncogenic K-Ras regulates the loading of specific miRNAs into sEVs by inhibiting the sorting of Argonaute2 (Ago2)-dependent miRNAs into sEVs, thereby influencing miRNA secretion in exosomes [21, 22]. Cancer-derived sEV-loaded miRNAs in the TME regulate genes in the surrounding cells by pairing to mRNAs of protein-coding genes to their posttranscriptional repression; thus, they induce malignant tumor progression by targeting a tumor suppressor [23, 24].

miR-494-3p is an onco-miRNA found in various cancers. Its expression has been found to be correlated with lung cancer progression in a K-Ras knock-in mouse model and with worse survival in lung cancer patients; moreover, it enhances the malignant progression of lung cancer cells by targeting phosphatase and tensin homolog (PTEN)/phosphoinositide 3-kinase (PI3K)/AKT signaling [25, 26]. miR-494-3p promotes the progression of various cancer cells, such as endometrial cancer, glioblastoma, prostate cancer, nasopharyngeal carcinoma, and hepatocellular carcinoma cells, by targeting PTEN or CXCR4 [27–31]. Furthermore, miR-494-3p regulates mitochondrial biogenesis and thermogenesis in beige adipocytes by targeting PPARγ coactivator1-α [32]; however, the detailed role of miR-494-3p in lung cancer progression, particularly in sEV-mediated intercellular communication during cancer progression in the TME, as well as the molecular mechanisms by which miR-494-3p regulates cancer progression in the TME, remain unclear.

In the present study, we demonstrated a key role for oncogenic Ras/syntenin-1 axis that can stimulate release of sEVs and loading of miR-494-3p into sEVs in human lung cancer cells, and define its role during cancer progression in sEVs-mediated intercellular communication between cancer cells and endothelial cells in TME.

## Results

### Syntenin-1 regulates the secretion of sEVs in human lung cancer cells

**Fig. 1:**
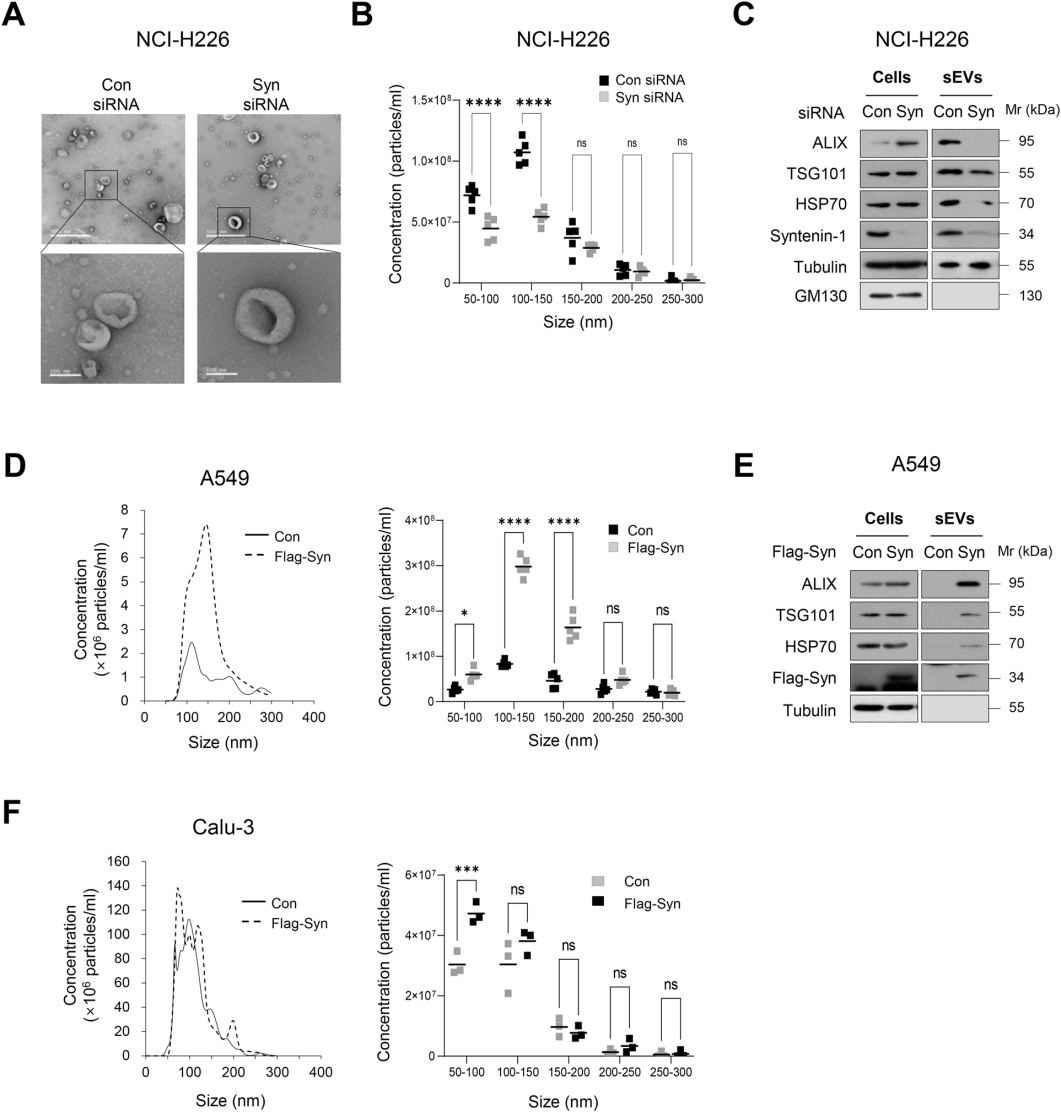
Syntenin-1 regulates the release of sEVs in lung cancer cells. **A** Representive TEM images of sEVs derived from control (Con) siRNA and syntenin-1 (Syn) siRNA transfected NCI-H226 cells. Scale bar, 500 nm (upper) and 100 nm (lower). **B** Nanoparticle tracking the size distribution of sEVs derived from NCI-H226 cells transfected with control (Con) siRNA or syntenin-1 (Syn) siRNA (*n* = 5). **C** Western blotting of sEV marker proteins in cell lysates and sEVs derived from NCI-H226 cells transfected with control (Con) siRNA or syntenin-1 (Syn) siRNA. **D** Nanoparticle tracking the size distribution of sEVs derived from A549 cells transfected with control (Con) or Flag-syntenin-1 (Flag-Syn) expression vector (*n* = 5). **E** Western blotting of sEV marker proteins in cell lysates and sEVs derived from A549 cells transfected with control (Con) or Flag-syntenin-1 (Flag-Syn) expression vector. **F** Nanoparticle tracking the size distribution of sEVs derived from Calu-3 cells transfected with control (Con) or Flag-syntenin-1 (Flag-Syn) expression vector (*n* = 3). *P < 0.05, ***P < 0.001, ****P < 0.0001.

We assessed the effect of syntenin-1 on the release of sEVs in human lung cancer cells exhibiting different syntenin-1 expression levels (Supplementary Fig. S1A). Transmission electron microscopy (TEM) analysis revealed no difference in structure and size between sEVs isolated from control and syntenin-1-knockdown NCI-H226 cells; moreover, the size of major sEV populations ranged from 50 to 200 nm (Fig. 1A and Supplementary Fig. S1B). Nanoparticle tracking analysis (NTA) revealed that the concentration of sEVs derived from syntenin-1-knockdown NCI-H226 cells was significantly decreased compared with that of sEVs derived from control cells (Fig. 1B). Syntenin-1 knockdown in NCI-H1299 cells also significantly decreased the concentration of sEVs ranging from 50 to 200 nm (Supplementary Fig. S1C). To further corroborate this effect of syntenin-1 on sEV secretion in lung cancer cells, we evaluated the effect of syntenin-1 on the expression of exosomal marker proteins, such as ALIX, TSG101, and HSP70. Syntenin-1 knockdown did not significantly decrease the expression of these marker proteins in whole cell lysates; however, it decreased the expression of these markers in sEVs purified from syntenin-1-knockdown NCI-H226 (Fig. 1C). The Golgi protein GM-130 was undetectable in sEVs isolated from control and syntenin-1-knockdown NCI-H226 cells. The overexpression of syntenin-1 in A549 cells markedly increased the concentration of sEVs ranging from 50 to 200 nm (Fig. 1D) and the expression of exosomal markers in the secreted EVs, but not in whole cell lysates (Fig. 1E). Furthermore, overexpression of syntenin-1 in Calu-3 cells increased the concentration of sEVs (Fig. 1F). Collectively, these findings indicated that syntenin-1 could regulate the biogenesis and secretion of sEVs in human lung cancer cells.

### Syntenin-1 mediates the oncogenic Ras-induced increase in sEV secretion in human lung cancer cells

**Fig. 2:**
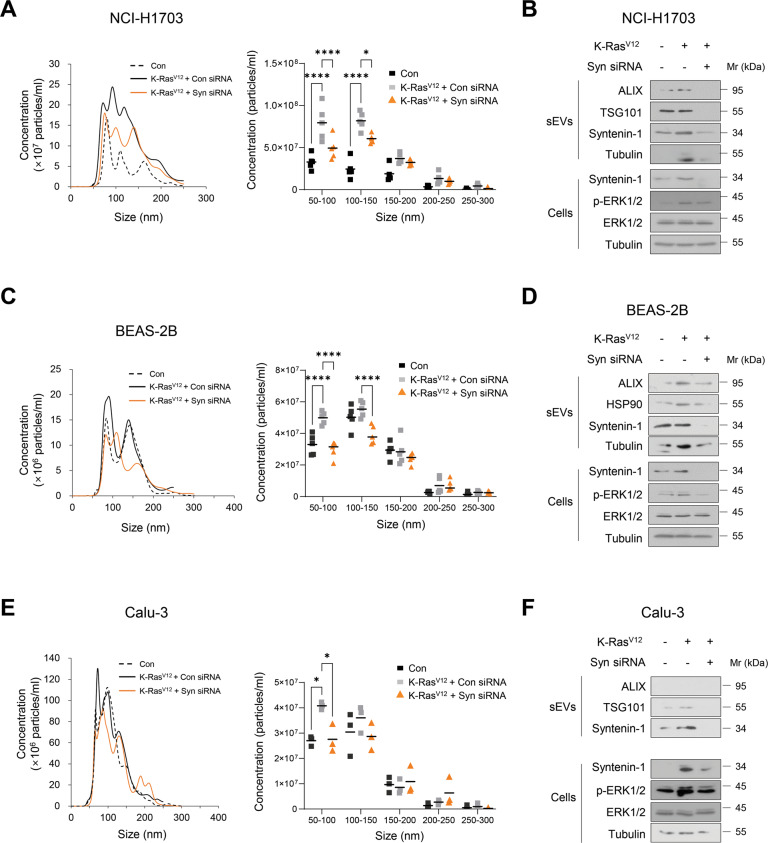
Syntenin-1 mediates oncogenic Ras-induced release of sEVs in lung cancer cells. **A** Nanoparticle tracking the size distribution of sEVs derived from K-Ras wild NCI-H1703 cells transfected with control (Con) vector, K-RasV12 expression vector (K-Ras^V12^) plus control siRNA (Con), or K-Ras^V12^ plus syntenin-1 (Syn) siRNA (*n* = 5). **B** Western blotting of proteins expressed in cell lysates and sEVs prepared from NCI-H1703 transfected with the indicated constructs. **C** Nanoparticle tracking the size distribution of sEVs derived from BEAS-2B cells transfected with control vector (Con), K-RasV12 expression vector (K-Ras^V12^) plus control (Con) siRNA, or K-Ras^V12^ plus syntenin-1 (Syn) siRNA (*n* = 5). **D** Western blotting of proteins expressed in cell lysates and sEVs prepared from BEAS-2B cells transfected with the indicated constructs. **E** Nanoparticle tracking the size distribution of sEVs derived from K-Ras wild Calu-3 cells transfected with control (Con), K-Ras^V12^ expression vector (K-Ras^V12^) plus control (Con) siRNA, or K-Ras^V12^ plus syntenin-1 (Syn) siRNA (*n* = 5). **F** Western blotting of proteins expressed in cell lysates and sEVs prepared from Calu-3 transfected with the indicated constructs. *P < 0.05, ****P < 0.0001.

Oncogenic Ras signaling plays a crucial role in the biogenesis, cargo loading, and release of sEVs [17, 21, 22]. Previous studies reported that oncogenic Ras induces syntenin-1 expression via JMJD3 histone K27 demethylase [33]. Therefore, we assessed the possibility that syntenin-1 is involved in oncogenic Ras-mediated release of sEVs in lung cancer cells. Transfection of K-RasV12 into K-Ras wild NCI-H1703 cells significantly increased the concentration of sEVs and expression of syntenin-1, ALIX, and TSG101 in sEVs; knockdown of syntenin-1 impaired the K-RasV12-induced increase in the concentration of sEVs and expression of exosomal markers in sEVs (Fig. 2A, B). We also determined the expression level of p-ERK as a marker for K-Ras activation after K-RasV12 transfection. Knockdown of syntenin-1 in K-Ras wild BEAS-2B or Calu-3 cells impaired the K-RasV12-induced increase in concentration of sEVs and expression of exosomal markers in sEVs (Fig. 2C–F). Moreover, knockdown of syntenin-1 in BZR cells, BEAS-2B cells transformed with H-RasV12, significantly reduced the concentration of secreted sEVs and the expression levels of exosomal marker proteins, including ALIX, TSG101, and HSP70, in sEVs (Supplementary Fig. S2A, B). Collectively, these findings suggested that syntenin-1 could play a pivotal role in oncogenic Ras-mediated biogenesis and secretion of sEVs in human lung cancer cells.

### sEVs derived from lung cancer cells promote cancer cell migration and tube formation in endothelial cells in a syntenin-1-dependent manner

**Fig. 3:**
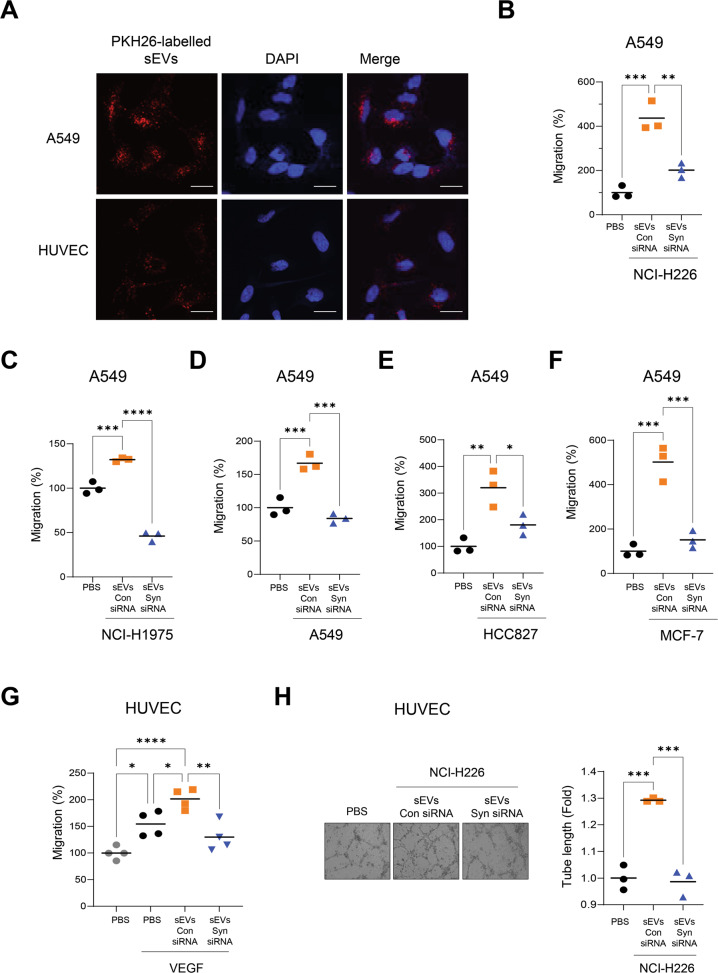
sEVs derived from syntenin-1-knockdown cancer cells abolished the stimulatory effects of sEVs on the migration of cancer and endothelial cells, and endothelial cell tube fromation. **A** sEVs purified from NCI-H226 cells were labelled with PKH26, and A549 cells and HUVECs were were incubated with PKH26-labelled sEVs for 12 h. Confocal images show uptake of sEVs by target cells. Cell nuclei were stained with DAPI. Scale bar, 20 μm. **B**–**F** sEVs were purified from the indicated human lung cancer cell lines that were transfected with control (Con) siRNA or syntenin-1 (Syn) siRNA. Transwell migration assays (*n* = 3) were performed to assess the migratory ability of A549 cells with or without the purified sEVs (10^9^ particles/ml). **G** HUVECs were treated with sEVs (10^9^ particles/ml) derived from NCI-H226 cells that were transfected with control (Con) siRNA or syntenin-1 (Syn) siRNA. Transwell migration assays (*n* = 4) were performed to assess the migratory ability of HUVECs with or without VEGF (10 ng/ml). **H** Tube formation assays (*n* = 3) for HUVECs were performed in the presence of sEVs (10^9^ particles/ml) derived from NCI-H226 cells that were transfected with control (Con) siRNA or syntenin-1 (Syn) siRNA. *P < 0.05, **P < 0.01, ***P < 0.001, ****P < 0.0001.

Herein, we determined whether sEVs enter target cells, such as endothelial and cancer cells. PKH26-labeled sEVs derived from NCI-H226 cells were internalized into endothelial cells and A549 cells (Fig. 3A), thereby suggesting that sEVs could be effectively internalized into target cells.

To assess the involvement of syntenin-1-mediated sEVs in cancer progression, we prepared sEVs from syntenin-1-knockdown cancer cell lines, such as NCI-H226, NCI-H1975, A549, HCC827, and MCF7 cells, and further evaluated their effects on the migration of A549 cells (Fig. 3B–F). The results indicated that treatment with sEVs derived from control cells significantly increased the migratory capacity of A549 cells, whereas sEVs derived from syntenin-1-knockdown cells diminished this effect (Fig. 3B–F), thereby suggesting that syntenin-1-mediated sEVs are crucial in inducing the migration of cancer cells. Consistently, migration of MDA-MB-231 or B16F10 cells was induced by the treatment of sEVs derived from control NCI-H226 cells, whereas sEVs derived from syntenin-1-knockdown NCI-H226 cells abolished this effect (Supplementary Fig. S3A, B). Furthermore, treatment with sEVs derived from syntenin-1-overexpressing MCF7 cells significantly increased the migratory potential of MDA-MB-231 cells (Supplementary Fig. S3C). Collectively, these findings indicate that syntenin-1-mediated sEVs may promote cancer cell migration.

Next, we investigated whether syntenin-1-mediated sEVs regulate angiogenesis. sEVs were purified from syntenin-1-knockdown NCI-H226 cells, and their effects on VEGF-induced migration and tube formation of endothelial cells was evaluated. sEVs derived from syntenin-1-knockdown cells did not significantly increase the VEGF-induced migration and tube formation of endothelial cells (Fig. 3G, H), thereby suggesting that syntenin-1-mediated sEVs in lung cancer cells could induce angiogenesis in the TME.

### Identification of miR-494-3p as a major onco-miRNA loaded into syntenin-1-mediated sEVs

**Fig. 4:**
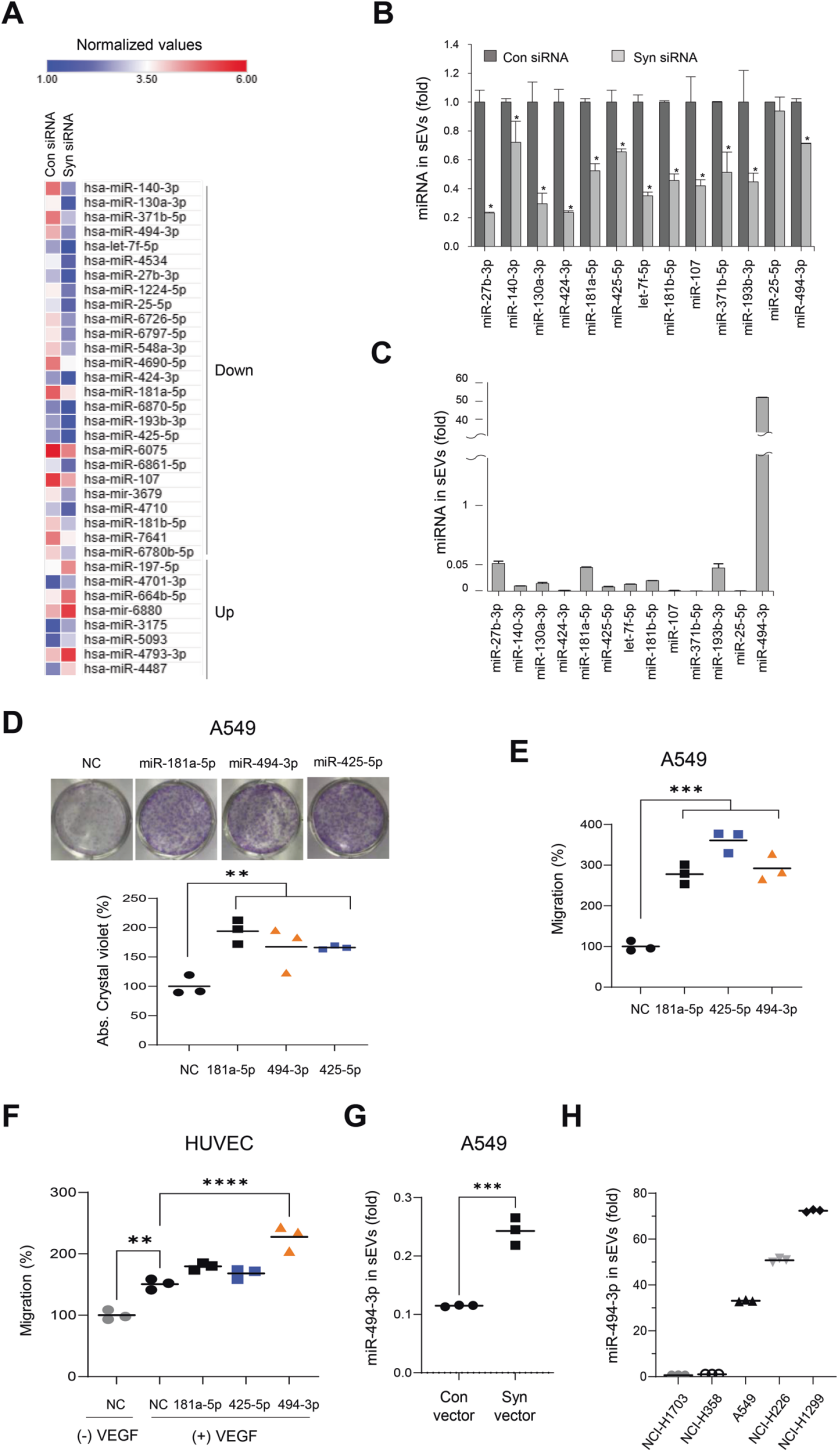
Identification of onco-miRNAs in syntenin-1-mediated sEVs in lung cancer cells. **A** Heatmap of the differentially expressed miRNAs (more than twofold) in sEVs derived from NCI-H226 cells that were transfected with control (Con) siRNA or syntenin-1 (Syn) siRNA. **B** Real-time qPCR analyses of the selected 13 downregulated miRNAs in sEVs derived from NCI-H226 cells that were transfected with control (Con) siRNA or syntenin-1 (Syn) siRNA. The results were normalized to U6 snRNA. **C** The expression levels of 12 downregulated miRNAs in sEVs derived from NCI-H226 cells determined by real-time qPCR analysis. The results were normalized to U6 snRNA. (**D** and **E**) A549 cells were transfected with 20 nM of control (NC), miR-181a-5p, miR-425-5p, or miR-494-3p, and colony formation (**D**) and transwell migration (**E**) assays were performed. **F** HUVECs were transfected with 20 nM of miR-181a-5p, miR-425-5p, or miR-494-3p and transwell migration assays were performed in the presence of VEGF (10 ng/ml). **G** sEVs were purified from A549 cells transfected with control (Con) or Flag-syntenin-1 (Syn) expression vector, and the expression level of miR-494-3p in sEVs was determined by real-time qPCR analysis. The results were normalized to U6 snRNA. **H** The expression level of miR-494-3p in sEVs derived from the indicated human lung cancer cell lines determined by real-time qPCR analysis. The results were normalized to U6 snRNA. *n* = 3, *P < 0.05, **P < 0.01, ***P < 0.001, ****P < 0.0001.

To assess the miRNAs that could be loaded into syntenin-1-mediated sEVs, the expression profiles of miRNAs were compared between sEVs isolated from control and syntenin-1-knockdown NCI-H226 cells. We found that the expression levels of 8 miRNAs were significantly upregulated, whereas those of 26 miRNAs were downregulated in sEVs derived from syntenin-1-knockdown NCI-H226 cells (Fig. 4A). Among the 26 downregulated miRNAs, 13 miRNAs were selected for further analysis. Moreover, qPCR analysis revealed that knockdown of syntenin-1 significantly decreased the expression levels of 12 miRNAs in sEVs, except miR-25-5p (Fig. 4B), whereas the cellular expression levels of these miRNAs revealed no significant change (Supplementary Fig. S4); of these, miR-27b-3p, miR-181a-5p, miR-193b-3p, and miR-494-3p were highly expressed in sEVs derived from NCI-H226 cells, as compared to other miRNAs (Fig. 4C). We evaluated the effects of these miRNAs on the proliferation and migration of cancer cells and VEGF-induced migration of endothelial cells. Notably, miR-181a-5p, miR-425-5p, and miR-494-3p stimulated the colony formation and migration of A549 cells (Fig. 4D, E) and miR-494-3p triggered the VEGF-induced migration of endothelial cells (Fig. 4F), thereby suggesting that miR-181a-5p, miR-425-5p, and miR-494-3p could be syntenin-1-dependent onco-miRNAs that were highly expressed in sEVs derived from NCI-H226 cells. We selected miR-494-3p for further analysis, since this miRNA was highly abundant in NCI-H226 cell-derived sEVs (Fig. 4C). Furthermore, overexpression of syntenin-1 into A549 cells increased the expression level of miR-494-3p in sEVs (Fig. 4G). In particular, the expression level of miR-494-3p in sEVs was significantly increased in human lung cancer cell lines that exhibited a higher level of syntenin-1 expression (Supplementary Figs. S1A, S5 and Fig. 4H). Collectively, these results suggested that syntenin-1 may regulate biogenesis and secretion of sEVs as well as miR-494-3p expression in sEVs; In addition, miR-494-3p could act as a major onco-miRNA in sEVs secreted from human lung cancer cells to induce cancer progression in the TME.

### Oncogenic Ras increases the secretion of miR-494-3p in sEVs via syntenin-1 in human lung cancer cells

**Fig. 5:**
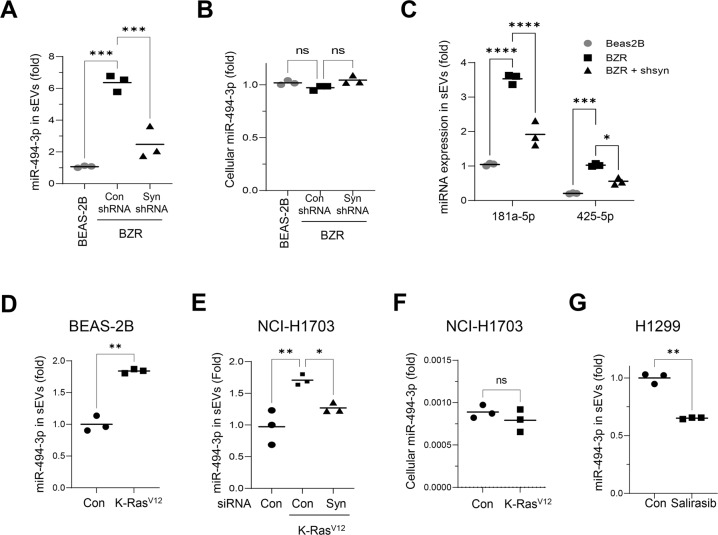
Syntenin-1 mediates oncogenic Ras-induced secretion of miR-494-3p in sEVs. **A**, **B** The expression level of miR-494-3p in sEVs (**A**) and cells (**B**) derived from BEAS-2B and BZR cells transfected with control (Con) shRNA or syntenin-1(Syn) shRNA was determined by real-time qPCR analysis. The results were normalized to U6 snRNA. **C** The expression levels of miR-181-5p and miR-425-5p in sEVs derived from BEAS-2B and BZR cells transfected with control (Con) shRNA or syntenin-1(Syn) shRNA were determined by real-time qPCR analysis. The results were normalized to U6 snRNA. **D** The expression level of miR-494-3p in sEVs derived from BEAS-2B cells transfected with control (Con) vector or K-RasV12 expression vector (K-Ras^V12^) was determined by real-time qPCR analysis. The results were normalized to U6 snRNA. **E** The expression level of miR-494-3p in sEVs derived from NCI-H1703 cells transfected with control (Con), K-RasV12 expression vector (K-Ras^V12^) plus control siRNA (Con), or K-Ras^V12^ plus syntenin-1 siRNA was determined by real-time qPCR analysis. The results were normalized to U6 snRNA. **F** The expression level of miR-494-3p in NCI-H1703 cells transfected with control (Con), K-RasV12 expression vector (K-Ras^V12^) was determined by real-time qPCR analysis. The results were normalized to U6 snRNA. **G** The expression level of miR-494-3p in sEVs derived from NCI-H1299 cells treated with salirasib (10 μM) was determined by real-time qPCR analysis. The results were normalized to U6 snRNA. *n* = 3, *P < 0.05, **P < 0.01, ***P < 0.001.

sEVs derived from H-RasV12-transformed BEAS-2B (BZR) cells expressed higher levels of miR-494-3p than those from BEAS-2B cells (Fig. 5A). In particular, knockdown of syntenin-1 in BZR cells decreased the expression level of miR-494-3p in sEVs; however, the cellular expression level of miR-494-3p was not significantly altered by H-RasV12 or knockdown of syntenin-1 (Fig. 5B). Moreover, the expression levels of miR-181a-5p and miR-425-5p increased in sEVs derived from BZR cells, whereas knockdown of syntenin-1 significantly suppressed H-RasV12-induced expression of these miRNAs in sEVs (Fig. 5C), thereby suggesting that the Ras/syntenin-1 axis could also regulate the loading and release of these two miRNAs into sEVs. Transfection of K-RasV12 into BEAS-2B cells also led to a significant increase in the expression of miR-494-3p in sEVs (Fig. 5D). Furthermore, transfection of K-RasV12 into K-Ras wild NCI-H1703 cells significantly increased the expression level of miR-494-3p in sEVs, whereas knockdown of syntenin-1 significantly impaired K-RasV12-induced miR-494-3p expression in sEVs (Fig. 5E); however, the cellular expression level of miR-494-3p in NCI-H1703 was not significantly altered by transfection of K-RasV12 (Fig. 5F). Consistently, the treatment of N-Ras mutant NCI-H1299 cells with salirasib, a pan Ras inhibitor, significantly decreased the expression level of miR-494-3p in sEVs (Fig. 5G). Collectively, these results suggested that oncogenic Ras could increase the expression level of miR-494-3p in sEVs through syntenin-1 in human lung cancer cells.

### miR-494-3p stimulates the proliferation and migration of endothelial and lung cancer cells

**Fig. 6:**
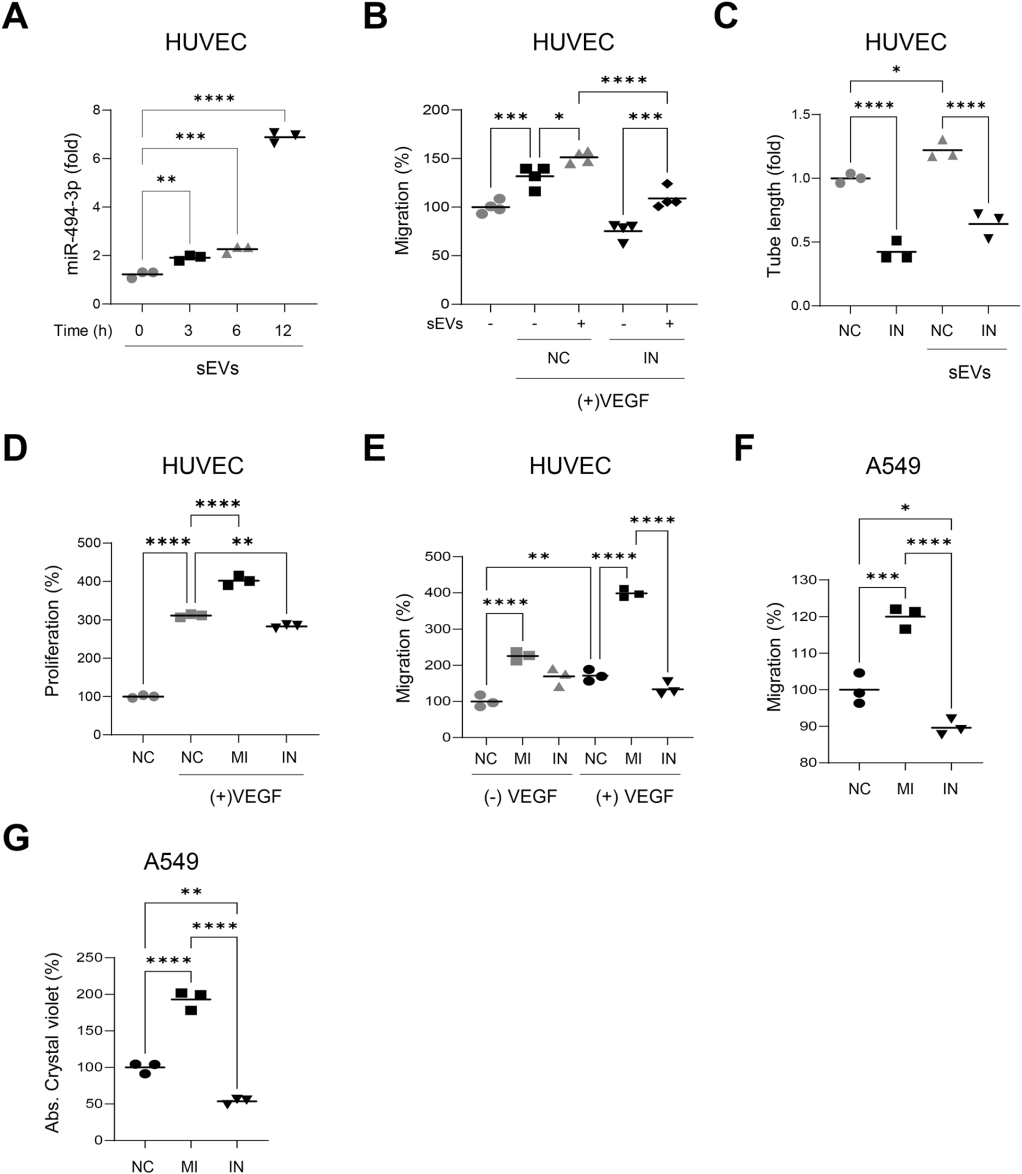
miR-494-3p stimulates the proliferation and migration of HUVECs and lung cancer cells. **A** HUVECs were incubated with sEVs purified from NCI-H226 cells for the indicated periods of time and the expression level of miR-494-3p in HUVECs was determined by real-time qPCR analysis. The results were normalized to U6 snRNA (*n* = 3). **B** HUVECs transfected with control (NC) or miR-494-3p inhibitor (IN) were incubated with or without sEVs purified from NCI-H226 cells. Transwell migration assays were performed to determine VEGF-induced migration of HUVECs (*n* = 4). **C** HUVECs transfected with control (NC) or miR-494-3p inhibitor (IN) were incubated with or without sEVs purified from NCI-H226 cells. Tube formation assays were performed (*n* = 3). **D**, **E** HUVECs were transfected with control (NC), miR-494-3p mimic (MI), or miR-494-3p inhibitor (IN). Cell proliferation (**D**) and transwell migration (**E**) assays were performed (*n* = 3). **F**, **G** A549 cells were transfected with control (NC), miR-494-3p mimic (MI), or miR-494-3p inhibitor (IN). Transwell migration (**F**) and colony formation (**G**) assays were performed (*n* = 3). *P < 0.05, **P < 0.01, ***P < 0.001, ****P < 0.0001.

Treatment of endothelial cells with sEVs derived from NCI-H226 cells increased the cellular expression level of miR-494-3p in a time-dependent manner (Fig. 6A), thereby indicating that miR-494-3p in sEVs was effectively delivered into the target cells. Moreover, the miR-494-3p inhibitor impaired sEV-induced migration of endothelial cells in the presence of VEGF (Fig. 6B and Supplementary Fig. S6A) and suppressed sEV-induced tube formation of endothelial cells (Fig. 6C). Transfection of the miR-494-3p mimic further increased VEGF-induced proliferation and migration of endothelial cells, whereas the miR-494-3p inhibitor reversed these effects (Fig. 6D, E and Supplementary Fig. S6B). These results suggested that miR-494-3p in sEVs may effectively increase sEV-mediated angiogenesis in the TME.

Next, we investigated the effect of miR-494-3p on the proliferation and migration of cancer cells. Notably, the miR-494-3p mimic significantly increased the migratory potential and colony-forming ability of A549 cells, whereas the miR-494-3p inhibitor significantly reversed these effects (Fig. 6F, G and Supplementary Fig. S6C), thereby indicating that the increased expression of miR-494-3p in cancer cells or sEVs might facilitate the proliferation and migration of cancer cells in the TME.

### miR-494-3p targets *PTPN12* and stimulates EGF and VEGF signaling

**Fig. 7:**
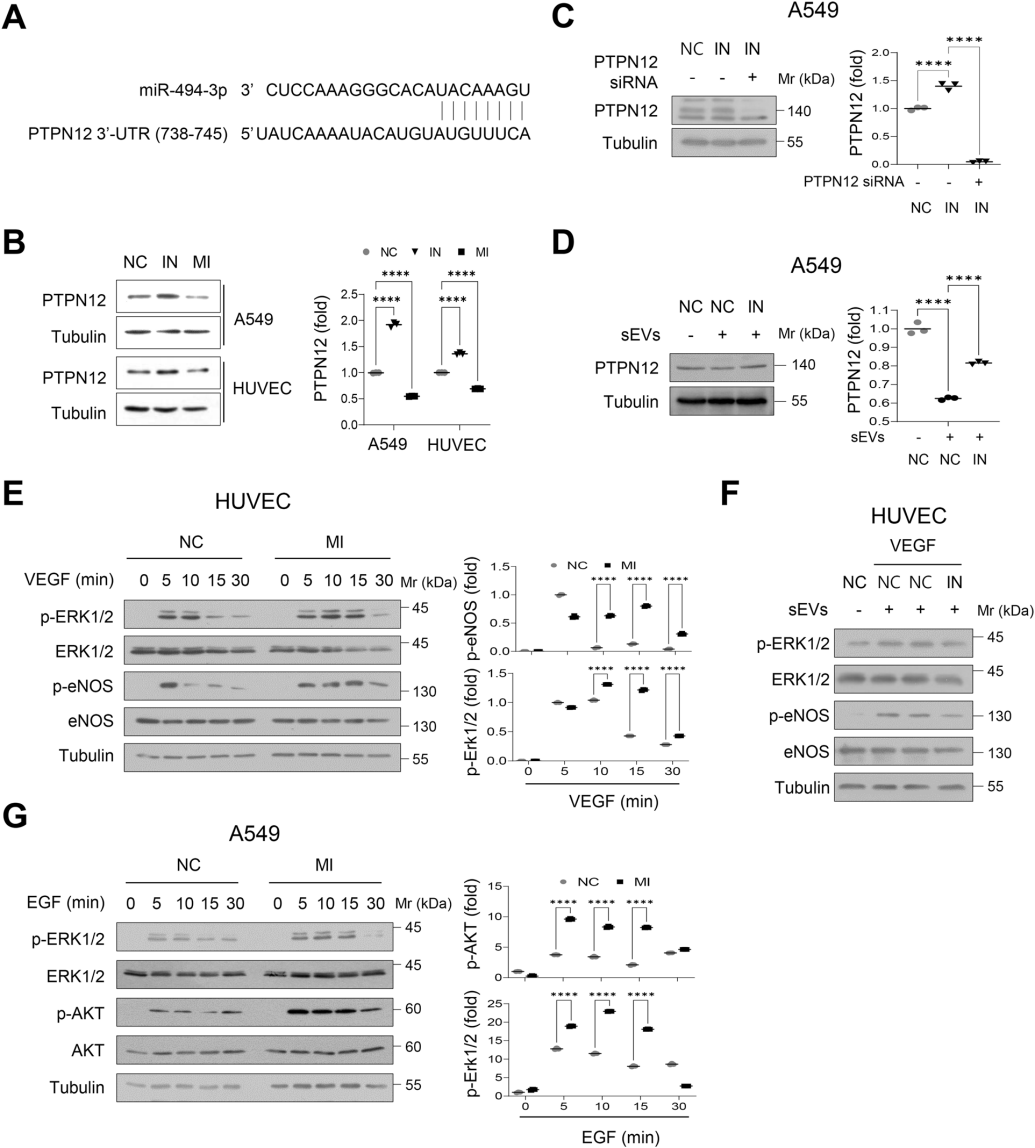
PTPN12 is a potential target of miR-494-3p. **A** The sequence alignment of hsa-miR-494-3p and 3′-UTR of PTPN12 mRNA. **B** Western blotting of PTPN12 in cell lysates derived from A549 cells and HUVECs transfected with control (NC), miR-494-3p mimic (MI), or miR-494-3p inhibitor (IN). **C** Western blotting of PTPN12 in cell lysates derived from A549 cells transfected with control (NC), miR-494-3p inhibitor (IN), or IN plus PTPN12 siRNA. **D** A549 cells transfected with control (NC) or miR-494-3p inhibitor (IN) were incubated with or without sEVs (10^9^ particles/ml) purified from NCI-H226 cells for 24 h. The expression level of PTPN12 was determined by Western blotting. **E** HUVECs were transfected with control (NC) or miR-494-3p mimic (MI), and were stimulated with VEGF (10 ng/ml) for the indicated periods of time. The expression levels of p-ERK1/2, p-ERK1/2, eNOS, and p-eNOS were determined by Western blotting. **F** HUVECs transfected with control (NC) or miR-494-3p inhibitor (IN) were incubated with or without sEVs (10^9^ particles/ml) purified from NCI-H226 cells, and were stimulated with VEGF (10 ng/nl) for 10 min. The expression levels of p-ERK1/2, p-ERK1/2, eNOS, and p-eNOS were determined by Western blotting. **G** A549 cells transfected with control (NC) or miR-494-3p mimic (MI), and were stimulated with EGF (10 ng/ml) for the indicated periods of time. The expression levels of p-ERK1/2, p-ERK1/2, AKT, and p-AKT were determined by Western blotting. The graphs represent densitometric analysis. *n* = 3, ****P < 0.0001.

By combining two bioinformatics tools (miRDB and TargetScan), PTPN12, also known as protein-tyrosine phosphatase (PTP)-PEST, was identified as a potential target mRNA of miR-494-3p (Fig. 7A). Importantly, the miR-494-3p mimic decreased the expression level of PTPN12 in A549 and endothelial cells, whereas its inhibitor reversed this effect (Fig. 7B). Knockdown of PTPN12 by siRNA inhibited the upregulation of PTPN12 induced by the miR-494-3p inhibitor (Fig. 7C). In particular, treatment with sEVs derived from NCI-H226 cells decreased the expression level of PTPN12 in endothelial cells, whereas the miR-494-3p inhibitor reversed this effect (Fig. 7D), thereby suggesting that miR-494-3p in sEVs is effectively delivered to the recipient cells and downregulates PTPN12 expression.

Next, we investigated whether miR-494-3p enhances the activation of signaling molecules induced by growth factors such as VEGF and EGF in endothelial and A549 cells. Transfection of endothelial cells with the miR-494-3p mimic significantly increased the VEGF-induced phosphorylation levels of ERK and eNOS (Fig. 7E), whereas its inhibitor reversed this effect (Supplementary Fig. S7A). Furthermore, transfection with the miR-494-3p inhibitor suppressed the sEV-induced increase in phosphorylation levels of ERK and eNOS in the presence of VEGF (Fig. 7F). Transfection of the miR‐494-3p mimic resulted in consistent upregulation of EGF-induced phosphorylation levels of AKT and ERK (Fig. 7G), whereas its inhibitor reversed this effect (Supplementary Fig. S7B). These findings suggested that miR-494-3p in cells or sEVs could target PTPN12 to promote the activation of signaling molecules mediated by multiple receptor tyrosine kinases, including EGFR and VEGFR2, in the TME.

### miR-494-3p in sEVs promotes tumor progression in vivo

**Fig. 8:**
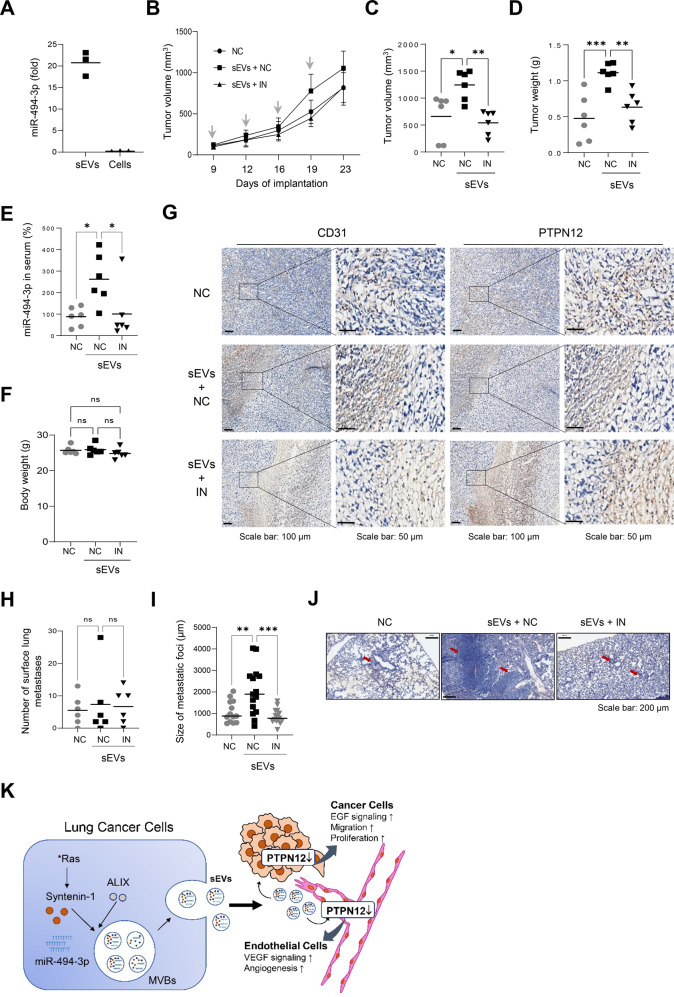
sEVs promote tumor growth of lung cancer cells, while miR-494-3p inhibitor suppresses it in vivo animal model. **A** The expression level of miR-494-3p in sEVs derived from Lewis lung carcinoma cells (LCC) determined by real-time qPCR analysis (*n* = 3). **B** C57BL/6 mice were subcutaneouly injected with LLC (10^6^ cells) into the right flank. When tumors reached a size of ~100 mm^3^, mice were intravenously injected with control miRNA (NC), sEVs derived from LLC plus NC, and sEVs derived from LLC plus miR-494-3p inhibitor (IN) every 3 or 4 days for 2 weeks. Mean ± SEM of tumor volume was shown (*n* = 6). **C**–**F** Tumor volume (**C**), tumor weight (**D**), the expression level of miR-494-3p in serum sEVs (**E**), and body weight (**F**) at the end of experiment (*n* = 6). **G** Tumor sections immunostained with anti-CD31 antibody and anti-PTPN12 antibody. Representative sections were taken from tumor tissue treated with control miRNA (NC), sEVs derived from LLC plus NC, and sEVs derived from LLC plus miR-494-3p inhibitor (IN). **H** The number of pulmonary surface metastases in the group treated with with control miRNA (NC), sEVs derived from LLC plus NC, and sEVs derived from LLC plus miR-494-3p inhibitor (IN). **I**, **J** Histological examination of pulmonary metastases. The size of metastatic foci more than 500 μm at longest diameter (**I**) and representative images of H&E-stained lung tissues (**J**). *P < 0.05, **P < 0.01, ***P < 0.001. **K** Working model of oncogenic Ras/syntenin-1 axis in regulating sEV-mediated tumor progression in lung cancer.

We conducted an experiment to assess the roles of sEVs and miR‐494-3p in tumor growth, metastasis, and angiogenesis in an animal model. Here, we used a syngeneic Lewis lung carcinoma (LLC) model. miR-494-3p was abundantly expressed in sEVs derived from LLC cells (Fig. 8A). Intravenous administration of sEVs derived from LLC cells into LLC-bearing mice significantly increased tumor growth and weight, and the expression level of miR-494-3p in serum sEVs, whereas treatment with the miR-494-3p inhibitor suppressed these effects (Fig. 8B–E); however, the miR‐494-3p inhibitor did not affect the body weight (Fig. 8F). Immunohistochemical staining revealed that the expression levels of CD-31 and PTPN12 in tumor tissues were significantly increased in the group treated with sEVs, whereas these levels were significantly decreased in the group treated with the miR-494-3p inhibitor (Fig. 8G). The number of lung surface metastases was not significantly increased in the group treated with sEVs (Fig. 8H); however, H&E staining of lung tissues revealed that the size of lung metastases was significantly increased in the group treated with sEVs (Fig. 8I, J). Furthermore, we examined the therapeutic potential of the miR-494-3p inhibitor in a xenograft model of NCI-H226 human lung cancer cells. Intra-tumoral administration of miR‐494-3p inhibitor had no effect on body weight; however, it significantly suppressed the growth of NCI-H226 xenograft and decreased the expression levels of PTPN12 and CD31 in xenograft tumors, as compared with that in the control (Supplementary Fig. S8). Collectively, these data indicate that miR-494-3p in sEVs may play a pivotal role in tumor growth, metastasis, and angiogenesis in vivo by targeting PTPN12.

## Discussion

The TME is crucial in development, growth, and metastasis of cancers during tumor progression. sEVs are abundant EVs containing numerous biological molecules, such as DNA, RNA, miRNA, and proteins, and act as a signaling complex, functional protein transporter, or genetic delivery device for mRNAs and miRNAs between cells in the TME [13, 34]. Moreover, cancer-derived sEVs remarkably affect the behavior of local or infiltrated stromal cells, which further supports tumor growth, angiogenesis, metastasis, drug-resistance, and immunosuppression of cancer cells [13, 34]. In the present study, we demonstrated that oncogenic Ras/syntenin-1 axis stimulates the release of sEVs and loading of onco-miRNAs, including miR-494-3p, into sEVs to promote the growth, migration, and metastasis of human lung cancer cells as well as tumor angiogenesis by targeting PTPN12; however, the suppression of oncogenic Ras or syntenin-1 inhibited these sEV-mediated effects (Fig. 8K). Therefore, syntenin-1 plays a pivotal role in malignant tumor progression of lung cancer cells in the TME by triggering the release of sEV-enriched miR-494-3p.

Syntenin-1 is overexpressed in various types of human cancers, and its expression level is correlated with poor prognosis in patients with cancer, including human lung and breast cancer [6, 7, 35, 36]. Presumably, the overexpression of syntenin-1 in certain cancer cells may promote the secretion of sEVs and affect the behavior of cancer cells and/or infiltrated stromal cells, thereby leading to malignant cancer cells; however, limited studies have reported the function of syntenin-1-regulated sEVs or miRNAs in sEVs. To the best of our knowledge, this is the first study to demonstrate the function of syntenin-1-mediated sEVs and miRNAs loaded into sEVs in the TME.

In the present study, loss of syntenin-1 reduced the biogenesis and secretion of sEVs in human lung cancer cells. Knockdown of syntenin-1 impaired the release of sEVs from cancer cells and downregulated the expression levels of exosomal markers, including TSG101, HSP70, and ALIX, in sEVs. In contrast, the overexpression of syntenin-1 reversed these effects, indicating that syntenin-1 could play a pivotal role in biogenesis and secretion of sEVs in lung cancer cells. The Ras signaling pathway triggers cancer progression. In particular, K-Ras mutations are related to poor survival in patients with non-small cell lung cancer and are detected in 15–20% of non-small cell lung cancer, particularly, 30–50% of adenocarcinomas [37, 38]. Emerging evidence suggests that Ras, including H-Ras and K-Ras, plays a pivotal role in biogenesis and release of exosomes as well as sorting of miRNAs to sEVs in various cancers [16, 17, 20]. We recently demonstrated that oncogenic Ras induces syntenin-1 expression in human lung cancer cells by activating KDM6B histone K3H27 demethylase [33]. The activation of K-Ras or H-Ras significantly triggered the release of sEVs and increased expression levels of several miRNAs, including miR-494-3p, in sEVs. In addition, knockdown of syntenin-1 reduced the oncogenic Ras-mediated release of sEVs and expression levels of these miRNAs in sEVs, thereby suggesting that syntenin-1 could act as one of the Ras signaling effectors for modulating the biogenesis and secretion of sEVs as well as for loading of specific onco-miRNAs, including miR-494-3p, into sEVs in order to induce tumor progression in the TME. However, the detailed mechanisms by which the Ras-syntenin-1 axis regulates the loading and release of onco-miRNAs into sEVs remain to be elucidated.

Increasing evidence suggests that sEV-derived miRNAs are involved in malignant tumor progression in various cancers and are likely to be used as potential therapeutic targets and valuable biomarkers for cancer treatment and diagnosis [24, 39, 40]. In the present study, we found that miR-494-3p was highly abundant in oncogenic Ras- and syntenin-1-dependent sEVs in human lung cancer cells, thereby suggesting that miR-494-3p in sEVs could be transferred to other cells and may modify the behavior of different cell types in the TME. miR-494-3p is an onco-miRNA that targets PTEN; however, the function of miR-494-3p in sEVs has not been fully elucidated. The present study revealed that the miR-494-3p mimic facilitated the migration and dissemination of cancer cells and VEGF-induced proliferation and migration of endothelial cells, while the miR-494-3p-inhibitor reversed these effects. Moreover, the miR-494-3p-inhibitor effectively inhibited the sEV-induced migration of cancer cells and VEGF-induced proliferation and migration of endothelial cells, suggesting that miR-494-3p in sEVs could function as a tumor promoter in an autocrine and paracrine manner in the TME. Furthermore, we identified PTPN12 as a potential target of miR-494-3p. PTPN12 is a tumor suppressor that inhibits multiple receptor tyrosine kinases, including epithelial growth factor receptor (EGFR) and HER2 in triple-negative breast cancer [41, 42]. PTPN12 positively regulates angiogenesis by stimulating endothelial cell migration [43]. We demonstrated that the miR-494-3p inhibitor impaired sEV-mediated downregulation of PTPN12, as well as EGF- and VEGF-induced activation of signaling molecules, whereas the miR-494-3p mimic reversed these effects. Moreover, intravenous administration of sEVs derived from lung cancer cells increased the expression level of PTPN12 and promoted tumor growth and angiogenesis in an animal model. In contrast, the miR-494-3p inhibitor significantly downregulated the expression of PTPN12 and suppressed tumor growth and angiogenesis, confirming that miR-494-3p in sEVs could significantly increase sEV-mediated cell–cell communication to induce malignant tumor progression in the TME.

In summary, our findings revealed that oncogenic Ras increased the release of sEVs by mediating syntenin-1 in human lung cancer cells. miR-494-3p was abundant in Ras- and syntenin-1-dependent sEVs and mediated sEV-induced migration and invasion of cancer cells and angiogenesis by targeting PTPN12 in the TME. Collectively, these findings suggest that syntenin-1 could serve as a downstream effector of the Ras pathway by inducing the release of sEVs and loading of miR-494-3p in sEVs to accelerate sEV-mediated malignant tumor progression in an autocrine and paracrine manner. Furthermore, these results demonstrate that miR-494-3p may be used as a potential target for human lung cancer therapy and diagnosis.

## Materials and methods

### Cells and cell culture

All cell lines used in this study were purchased from the American Type Culture Collection (ATCC, Manassas, VA, USA). A549, NCI-H1975, NCI-H226, HCC827, MCF-7, and BEAS-2B, an immortalized human bronchial epithelial cell line, cells were maintained in RPMI-1640 medium supplemented with 10% heat-inactivated fetal bovine serum (FBS, BioWest, Riverside, MO, USA) and 1% penicillin/streptomycin (Gibco, Waltham, MA, USA). BZR, viral H-Ras (H-Ras^G12V^) transformed BEAS-2B cells, LLC and MDA-MB-231 cells were cultured in DMEM supplemented with 10% FBS and 1% penicillin/streptomycin. Human umbilical vein endothelial cells (HUVECs) were purchased from ATCC and were cultured in M199 medium containing 20% heat-inactivated FBS, 1% penicillin/streptomycin, 15 μg/ml endothelial cell growth supplement (ECGS, Corning, NY, USA) and 5 units/ml of heparin (Sigma-Aldrich, St Louis, MO, USA). All cells were maintained in a humidified 5% CO_2_ atmosphere at 37 °C.

### Reagents and antibodies

Anti-Akt (cat. no. 9272), anti-phospho-Akt (cat. no. 9271), anti-Erk (cat. no. 4695), anti-phospho-Erk (cat. no. 9101), anti-tubulin (cat. no. 3878) antibodies were purchased from Cell signaling Technology (Danvers, MA, USA). Anti-eNOS (cat. no. 610296), and anti-phospho-eNOS (cat. no. 612392) antibodies were purchased from BD Biosciences (San Diego, CA, USA). Anti-Syntenin-1 (cat. no. sc-515507), anti-ALIX (cat. no. sc-53540), anti-TSG101 (cat. no. sc-7964), anti-HSP90 (cat. no. sc-13119), anti-HSP70 (cat. no. sc-32239), and anti-PTPN12 (cat. no. sc-271351) antibodies were purchased from Santa Cruz Biotechnology (Santa Cruz, CA, USA). Recombinant human vascular endothelial growth factor (VEGF, cat. no. V7259), recombinant human epidermal growth factor (EGF, cat. no. E5036) and salirasib (cat. no. S7684) were purchased from Sigma-Aldrich.

### Plasmids, RNA interference, and small hairpin RNA

The expression vectors for Flag-Syntenin-1, K-RasV12, and H-RasV12 were previously described [8, 33]. The shRNA-producing plasmids (cat. no. TL309594) were constructed according to the manufacturer’s protocol (OriGene, Rockville, MD, USA). siRNAs for syntenin-1 (cat. no. SR321723) and scramble control (cat. no. SR30005) were purchased from OriGene. miR-494-3p mimic (5′-UGAAACAUACACGGGAAACCUC-3′), miR-494-3p inhibitor (5′-ACUUUGUAUGUGCCCUUUGGAG-3′), and miRNA negative control (cat. no. SMC-2001) were purchased from Bioneer (Daejeon, Republic of Korea). Transfections were performed using Lipofectamine and RNAiMAX lipofectamine according to the manufacturer’s instruction (Thermo Fisher Scientific, Waltham, MA, USA).

### sEVs isolation and nanoparticle tracking analy**sis (NTA)**

The conditioned medium was collected from 4 × 10^6^ cells after 24 h incubation in serum-free medium and used for sEVs isolation by ultracentrifugation as previously described [44]. Briefly, the collected conditioned medium was centrifuged at 300 × *g* for 15 min at room temperature to remove cellular debris and subsequently centrifuged at 10,000 × *g* for 30 min at 4 °C. The supernatant was centrifuged at 100,000 × *g* for 70 min at 4 °C and then the precipitated sEVs were washed with phosphate-buffered saline (PBS). The precipitated sEVs were suspended with PBS and then centrifuged at 100,000 × *g* for 70 min at 4 °C again. The supernatant was discarded, and the precipitated sEVs were used for further experiments. Size distribution and concentration of sEVs were analyzed by NTA using NanoSight NS300 (Malvern Panalytical, Malvern, UK). The purified sEVs were injected into the laser chamber using a 1 mL syringe and 30 s recordings were performed. The mode, mean size, and concentration of sEVs were determined by NTA 3.0 software.

### Transmission electron microscopy

The purified sEVs were diluted with PBS for negative staining. Twenty μL of sEVs in PBS were applied to the carbon-coated grid, which had been previously glow-discharged (Harrick Plasma, USA) for 3 min in air, followed by negative staining with 2% uranyl acetate. The prepared grids were examined by TEM using a JEM 2100 F model microscope (JEOL, Japan; Korea Basic Science Institute Chuncheon Center, Chuncheon, Republic of Korea) operating at 200 kV. The images were acquired with the Ganta One view camera.

### In vitro sEVs uptake assay

sEVs derived from H226 cells (4 × 10^6^ cells) were labeled with PKH26 (cat. no. PKH26GL) according to the manufacturer’s protocol (Sigma-Aldrich). Briefly, PKH26 dye was diluted in 100 μL diluent C to a final concentration of 8 μM (dye solution). Then, 10^7^ particles of sEVs (NTA analysis) in 20 μL PBS were diluted with 80 μL diluent C, added to the dye solution, and incubated for 5 min while mixed with gentle pipetting. Excess dye was bound with 100 μL 10% BSA in PBS. Then the sEVs were diluted to 1 mL with DPBS and pelleted by ultracentrifugation at 120,000 × *g* for 1 h 10 min at 4 °C (Beckman Coulter). The pellet was gently resuspended in 50 μL PBS, and A549 and HUVECs (5 × 10^4^ cells/mL) were incubated with 10 μL of PKH26-labeled sEVs (2 × 10^6^ particles) at 37 °C with 5% CO_2_. After incubation, cells were washed twice with PBS and fixed with 4% paraformaldehyde for 10 min at room temperature. The cells were washed twice with PBS, 4′,6-diamidino-2-phenylindole dihydrochloride (DAPI, Sigma-Aldrich) were added, and the cells were incubated for 15 min at room temperature. Cellular uptake of cells-derived sEVs was observed under a confocal laser microscope.

### Quantification of miRNA by real-time quantitative polymerase chain reaction (qPCR)

Total RNAs were isolated using the TRIzol reagent according to the manufacturer’s instructions (Thermo Fisher Scientific, Waltham, MA, USA). The expression levels of miRNAs were quantified by RT- qPCR using the miScript PCR System (Qiagen, Valencia, CA, *USA*). cDNA was synthesized using the miScript II RT kit according to the manufacturer’s instructions (Qiagen). Real-time qPCR was performed on a StepOne Real-time PCR System (Applied Biosystems, Foster City, CA, USA) using 2 × Fast Q-PCR Master mix (SMOBiO, Hsinohu City, Taiwan). U6 small nuclear RNA (snRNA) was amplified as an internal control. Primer sequences used in this study were listed in Supplementary Table S1.

### Cell migration assays

Cell migration assays were performed using the Boyden chamber assay as previously described [8, 33]. Briefly, cells were seeded in triplicate at a density of 5 × 10^4^ cells/well (cancer cells) or 2 × 10^4^ cells/well (HUVECs) onto the upper chambers with 200 μL of the medium containing 0.5% FBS, and the upper chambers were placed into the lower chambers of 24-well culture plate containing 800 μL of the medium containing 10% FBS. sEVs (10^9^ particles/ml) isolated from control or syntenin-1-knockdown cells were added to the upper chamber. In certain experiments, VEGF (40 ng/ml) was added to the lower chamber. Following incubation at 37 °C, migrated cells on lower surface of the filter were fixed with methanol. The cells were then stained with hematoxylin and eosin (H&E), and then counted in at least five randomly selected microscopic fields (×100) per filter.

### miRNA array

sEVs were purified from control or syntenin knockdown NCI-H226 cells and miRNA array was performed by Macrogen Inc. (Seoul, Republic of Korea). The miRNA Microarray system with Affymetrix GeneChip^®^ 4.0 array containing 2578 human mature miRNA oligonucleotide probes was used according to the manufacturer’s recommended protocol (Santa Clara, CA, USA). Raw data were extracted automatically in Affymetrix data extraction protocol using the software provided by Affymetrix GeneChip^®^ Command Console^®^ Software (AGCC). The CEL files import, miRNA level RMA + DABG-All analysis and result export using Affymetrix^®^ Power Tools (APT) Software. Array data were filtered by probes annotated species. The comparative analysis between test sample and control sample was carried out using fold-change. For a significant DEmiRNA set, hierarchical cluster analysis was performed using complete linkage and Euclidean distance as a measure of similarity. All Statistical test and visualization of differentially expressed genes was conducted using R statistical language v.3.3.2 (https://www.r-project.org).

### Matrigel tube formation and colony formation assays

Matrigel (150 μL) was coated onto the wells of 48-well plate, and polymerized for 1 h at 37 °C. HUVECs (1 × 10^5^ cells/well) were seeded on polymerized Matrigel and cultured with or without sEVs (10^9^ particles/ml). Tubular networks in each well were photographed using CKX41 inverted microscope (Olympus, Tokyo Japan). For colony formation assay, A549 cells were cultured into 24-well plate (500 cells/well) for 24 h, and were treated with sEVs (10^9^ particles/ml). After 2 weeks, cells were stained with crystal violet in 40% MeOH for 10 min, then washed with PBS three times. The crystal violet dye was solubilized with 1 ml of 10% acetic acid and the absorbance of the solution was measured at a wavelength of 570 nm.

### Western blotting

Cells and sEVs were lysed in lysis buffer [50 mM Tris-HCl, pH 7.4, 150 mM NaCl, 1 mM EDTA, 5 mM sodium orthovanadate, 1% NP-40 and protease inhibitor cocktail (BD Biosciences)] and the lysates were centrifuged at 15,000 *rpm* for 15 min at 4 °C. Equal amounts of the resulting protein extracts were separated by SDS-polyacrylamide gel electrophoresis and transferred onto a Hybond-P membrane (Sigma-Aldrich). The membrane was blocked with 5% skim milk or BSA, and then incubated for overnight with the primary antibody at 4 °C. After washing, the membrane was incubated with the appropriate secondary antibody conjugated to horseradish peroxidase. The signal was detected using the enhanced chemiluminescence system (DoGenBio, Seoul, Republic of Korea). Quantitation was carried out using ImageJ Software (NIH, Bethesda, MD, USA).

### Animal experiments

Six-week-old male C57BL6 mice were purchased from DBL (Chungbug, Republic of Korea). Lewis lung carcinoma (LLC) cells (10^6^) were subcutaneously injected into the right flank of the mice and tumor volume was monitored by using the formula: tumor volume = 0.5 × length × width^2^, where length represents the longest tumor diameter and width represents the perpendicular tumor diameter. When average tumor volume reached ~100 mm^3^, the mice randomly divided into the following three groups: control group (*n* = 6) receiving negative control miRNA alone (NC, 0.03 nmol/mouse); sEVs group (*n* = 6) receiving sEVs (10^9^ particles/mouse) alone derived LLC cells plus NC: sEVs plus inhibitor group (*n* = 6) receiving sEVs (10^9^ particles/mouse) derived LLC cells and miR-494-3p inhibitor (IN, 0.03 nmol/mouse). Animals were treated intravenously via the tail vein with NC alone, sEVs plus NC, or sEVs plus IN twice (every 3 or 4 days) a week for 2 weeks. NC and IN were incubated with sEVs for 30 min before tail-vein injection. Tumor volume was measured twice (every 3 or 4 days) a week. At the end of experiment, all tumor tissues, lungs, and blood were collected. The number of pulmonary metastases was determined by counting the number of surface metastases. Then The lungs were fixed with formalin, embedded in OCT compound, sectioned, and stained with H&E. To evaluate anti-tumor effect of miR-494-3p inhibitor in NCI-H226 xenograft model, six-week-old female athymic BALB/c nude mice (DBL) were subcutaneously injected with NCI-H226 cells (2 × 10^6^). When average tumor volume reached ~100 mm^3^, NC or IN was administered via intra-tumoral injection twice (every 3 or 4 days) a week for 2 weeks. At the end of experiment, all tumor tissues were collected and fixed in formalin for immunohistochemistry.

### Immunohistochemistry

Tumor tissues were fixed in 4% paraformaldehyde for 12 h. The samples were incubated in 30% sucrose for 12 h, and then embedded in OCT compound and cut to 5 µm sections. The slides were incubated with specific antibodies for anti-CD31 (1:200, cat. no. 553370, BD Biosciences) and anti-PTPN12 (1:100, cat. no. sc-271351, Santa Cruz Biotechnology). followed by HRP-conjugated anti-mouse or anti-rabbit IgG using the cell & tissue staining kit (R&D systems). The reaction was then developed with DAB substrate.

### Statistical analysis

Data are expressed as the mean ± standard deviation (SD). All statistical analysis to determine significance between groups was performed using GraphPad Prism 9 (San Diego, CA, USA) using a Two-Way ANOVA with post-hoc tukey’s multiple comparisons test.

## Supplementary information

Supplementary Table S1

Supplementary Figure S1

Supplementary Figure S2

Supplementary Figure S3

Supplementary Figure S4

Supplementary Figure S5

Supplementary Figure S6

Supplementary Figure S7

Supplementary Figure S8

Reproducibility checklist

## Data Availability

The data that support the findings of this study are available from the corresponding author upon reasonable request.
