# Supplementary material for: Syntenin-1-mediated small extracellular vesicles promotes cell growth, migration, and angiogenesis by increasing onco-miRNAs secretion in lung cancer cells
Source: Cell Death Dis. 2022 Feb 8;13(2):122. doi: 10.1038/s41419-022-04594-2 (PMC8826407; doi:10.1038/s41419-022-04594-2)
Supplement: Supplementary file 1 — Supplementary Table S1 [file 41419_2022_4594_MOESM1_ESM.pdf]

Supplementary Table S1. List of qPCR primers for miRNAs.

miRNA Name	Forward primer (5`- to -3`)
U6 snRNA	CGCAAGGATGACACGCAAATTC
mmu-miR-494-3p	TGAAACATACACGGGAAACCTC
hsa-let-7f-5p	TGAGGTAGTAGATTGTATAGTT
hsa-miR-107	AGCAGCATTGTACAGG GCTATCA
hsa-miR-130a-3p	CAGTGCAATGTTAAAAGGGCAT
hsa-miR-140-3p	TACCACAGGGTAGAACCACGG
hsa-miR-181a-5p	AACATTCAACGCTGTCG GTGA GT
hsa-miR-181b-5p	AACATTCATTGCTGTCGGTGGGT
hsa-miR-193b-3p	AACTGGCCCTCAAAGTCCCGCT
hsa-miR-25-5p	AGGCGGAGACTTGGG CAATTG
hsa-miR-27b-3p	TTCACAGTGGCTAAGTTCT
hsa-miR-424-3p	CAAAACGTGAGGCGCTGCTAT
hsa-miR-425-5p	AATGACACGATCACTCCCGT TGA
hsa-miR-371b-5p	ACTCAAAAGATGGCGGCACTTT
hsa-miR-494-3p	TGAAACATACACGGGAAACCTC
