# Supplementary material for: Syntenin-1-mediated small extracellular vesicles promotes cell growth, migration, and angiogenesis by increasing onco-miRNAs secretion in lung cancer cells
Source: Cell Death Dis. 2022 Feb 8;13(2):122. doi: 10.1038/s41419-022-04594-2 (PMC8826407; doi:10.1038/s41419-022-04594-2)
Supplement: Supplementary file 2 — Supplementary Figure S1 [file 41419_2022_4594_MOESM2_ESM.pdf]

Supplementary Figure S1

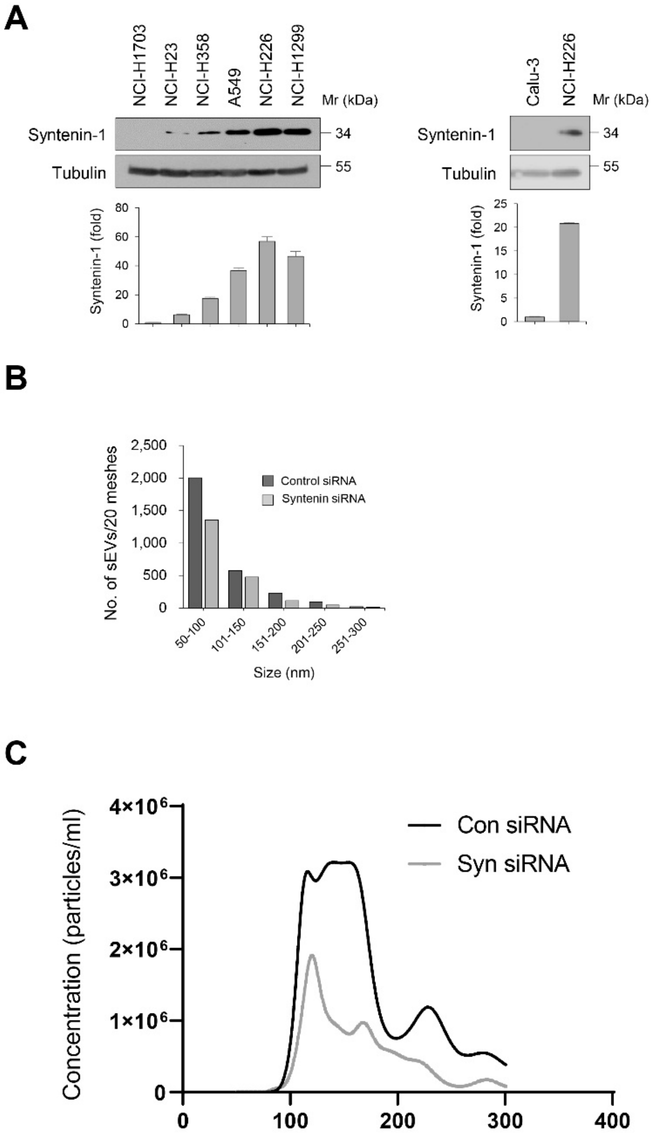

Supplementary Figure S1. (**A**) Western blotting of syntenin-1 in cell lysates derived from the indicated human lung cancer cell lines. A graph represnts densitometric analysis of western blot. (**B**) Size distribution of TEM images of sEVs derived from control (Con) siRNA and syntenin-1 (Syn) siRNA transfected NCI-H226 cells. (**C**) Nanoparticle tracking the size distribution of sEVs derived from NCI-H1299 cells transfected with control (Con) siRNA or syntenin-1 (Syn) siRNA (n = 3).
