# Supplementary material for: Syntenin-1-mediated small extracellular vesicles promotes cell growth, migration, and angiogenesis by increasing onco-miRNAs secretion in lung cancer cells
Source: Cell Death Dis. 2022 Feb 8;13(2):122. doi: 10.1038/s41419-022-04594-2 (PMC8826407; doi:10.1038/s41419-022-04594-2)
Supplement: Supplementary file 3 — Supplementary Figure S2 [file 41419_2022_4594_MOESM3_ESM.pdf]

Supplementary Figure S2

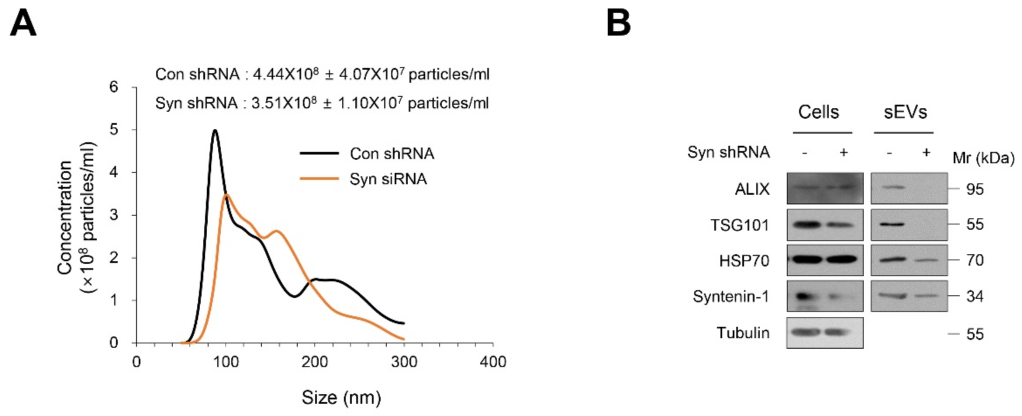

Supplementary Figure S2. **Syntenin-1 mediates oncogenic H-Ras-induced release of sEVs in BZR cells.** (**A**) Nanoparticle tracking the size distribution of sEVs derived from BZR cells transfected with control (Con) shRNA or syntenin-1 (Syn) shRNA (n = 3). (**B**) Western blotting of sEV marker proteins in cell lysates and sEVs derived from BZR cells transfected with control (Con) shRNA or syntenin-1 (Syn) siRNA.
