# Supplementary material for: Syntenin-1-mediated small extracellular vesicles promotes cell growth, migration, and angiogenesis by increasing onco-miRNAs secretion in lung cancer cells
Source: Cell Death Dis. 2022 Feb 8;13(2):122. doi: 10.1038/s41419-022-04594-2 (PMC8826407; doi:10.1038/s41419-022-04594-2)
Supplement: Supplementary file 4 — Supplementary Figure S3 [file 41419_2022_4594_MOESM4_ESM.pdf]

Supplementary Figure S3

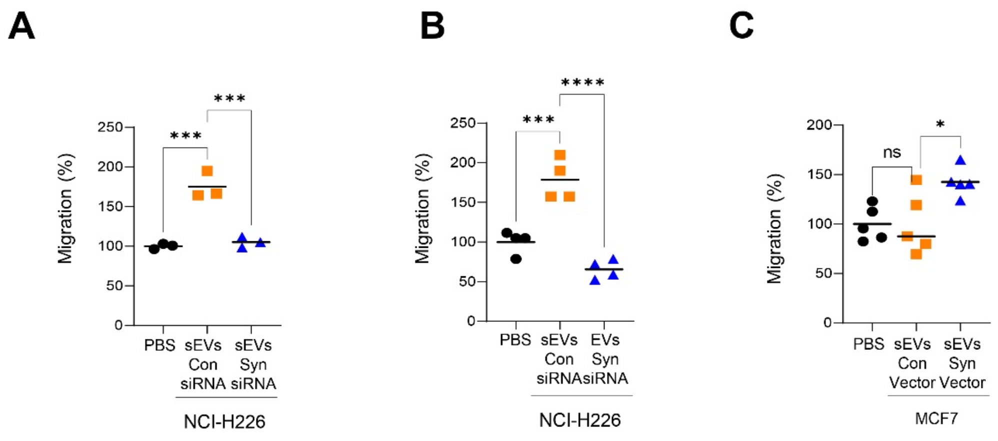

Supplementary Figure S3. Knockdown of syntenin-1 diminishes the stimulatory effect of sEVs on the migration of various cancer cells. (**A** and **B**) sEVs were purified from NCI-H226 cells that were transfected with control (Con) siRNA or syntenin-1 (Syn) siRNA. Transwell migration assays were performed to assess the migratory ability of MDA-MB-231 (**A**) and B16F10 (**B**) cells with or without the purified sEVs (10^9 particles/ml). (**C**) sEVs were purified from MCF-7 cells transfected with control (Con) or Flag-syntenin-1 (Syn) expression vector. Transwell migration assays were performed to assess the migratory ability of MDA-MB-231 cells with or without the purified sEVs (10^9 particles/ml). n = 3, **P*<0.05, ***P*<0.01, ****P*<0.001.
