# Supplementary material for: Syntenin-1-mediated small extracellular vesicles promotes cell growth, migration, and angiogenesis by increasing onco-miRNAs secretion in lung cancer cells
Source: Cell Death Dis. 2022 Feb 8;13(2):122. doi: 10.1038/s41419-022-04594-2 (PMC8826407; doi:10.1038/s41419-022-04594-2)
Supplement: Supplementary file 5 — Supplementary Figure S4 [file 41419_2022_4594_MOESM5_ESM.pdf]

Supplementary Figure S4

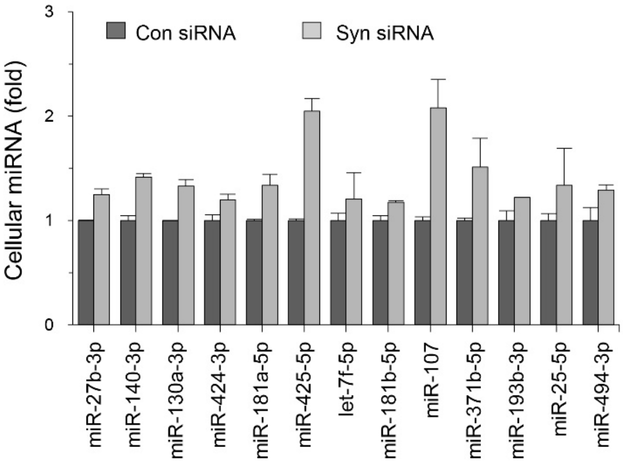

Supplementary Figure S4. Real-time qPCR analysis of the selected 13 down-regulated miRNAs in syntenin-1 knockdown NCI-H226 cells. The results were normalized to U6 snRNA (n = 3).
