# Supplementary material for: Syntenin-1-mediated small extracellular vesicles promotes cell growth, migration, and angiogenesis by increasing onco-miRNAs secretion in lung cancer cells
Source: Cell Death Dis. 2022 Feb 8;13(2):122. doi: 10.1038/s41419-022-04594-2 (PMC8826407; doi:10.1038/s41419-022-04594-2)
Supplement: Supplementary file 6 — Supplementary Figure S5 [file 41419_2022_4594_MOESM6_ESM.pdf]

Supplementary Figure S5

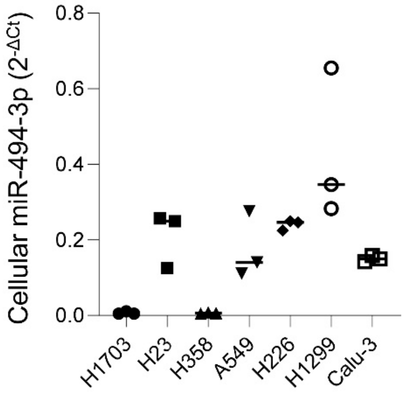

Supplementary Figure S5. The cellular expression level of miR-494-3p in the indicated human lung cancer cell lines was determined by real-time qPCR analysis. The results were normalized to U6 snRNA (n = 3).
