# Supplementary material for: Syntenin-1-mediated small extracellular vesicles promotes cell growth, migration, and angiogenesis by increasing onco-miRNAs secretion in lung cancer cells
Source: Cell Death Dis. 2022 Feb 8;13(2):122. doi: 10.1038/s41419-022-04594-2 (PMC8826407; doi:10.1038/s41419-022-04594-2)
Supplement: Supplementary file 7 — Supplementary Figure S6 [file 41419_2022_4594_MOESM7_ESM.pdf]

## Supplementary Figure S6

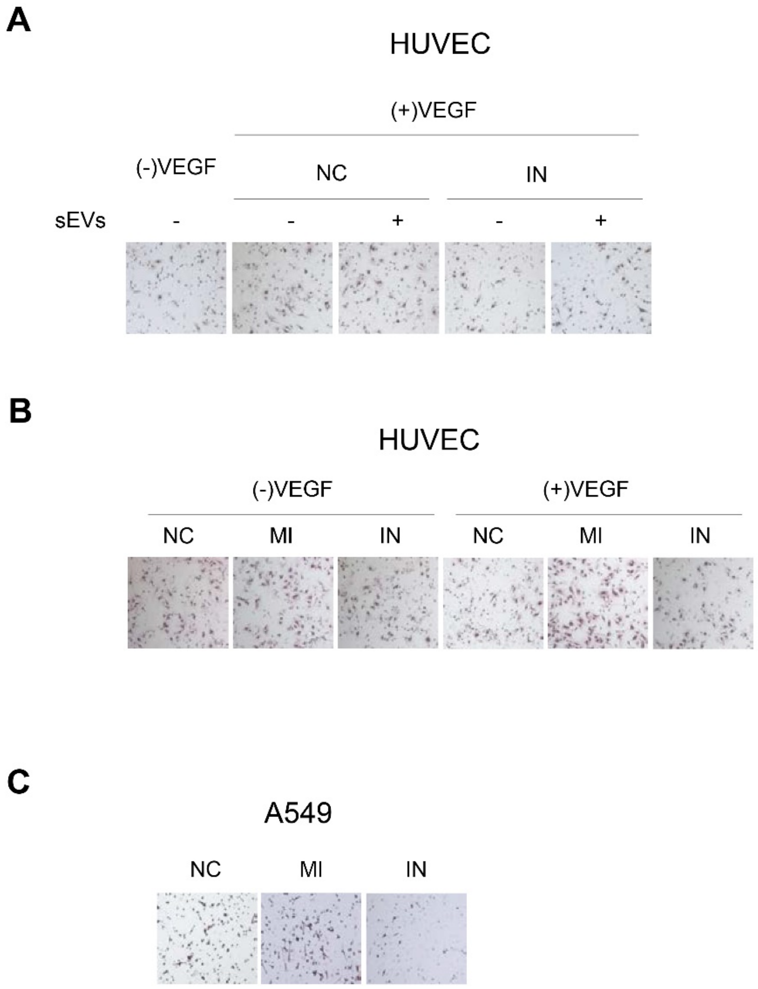


**Supplementary Figure S6. Representative Images of migration assays corresponding to Figure 6.** (**A**) HUVECs transfected with control (NC) or miR-4Representative images of Figure 6B. 94-3p inhibitor (IN) were incubated with or without sEVs purified from NCI-H226 cells. Transwell migration assays were performed to determine VEGF-induced migration of HUVECs. (**B**) Representative images of Figure 6E. HUVECs were transfected with control (NC), miR-494-3p mimic (MI), or miR-494-3p inhibitor (IN). Transwell migration assays were performed. (**C**) Representative images of Figure 6F. A549 cells were transfected with control (NC), miR-494-3p mimic (MI), or miR-494-3p inhibitor (IN). Transwell migration assays were performed.
