# Supplementary material for: Syntenin-1-mediated small extracellular vesicles promotes cell growth, migration, and angiogenesis by increasing onco-miRNAs secretion in lung cancer cells
Source: Cell Death Dis. 2022 Feb 8;13(2):122. doi: 10.1038/s41419-022-04594-2 (PMC8826407; doi:10.1038/s41419-022-04594-2)
Supplement: Supplementary file 8 — Supplementary Figure S7 [file 41419_2022_4594_MOESM8_ESM.pdf]

Supplementary Figure S7

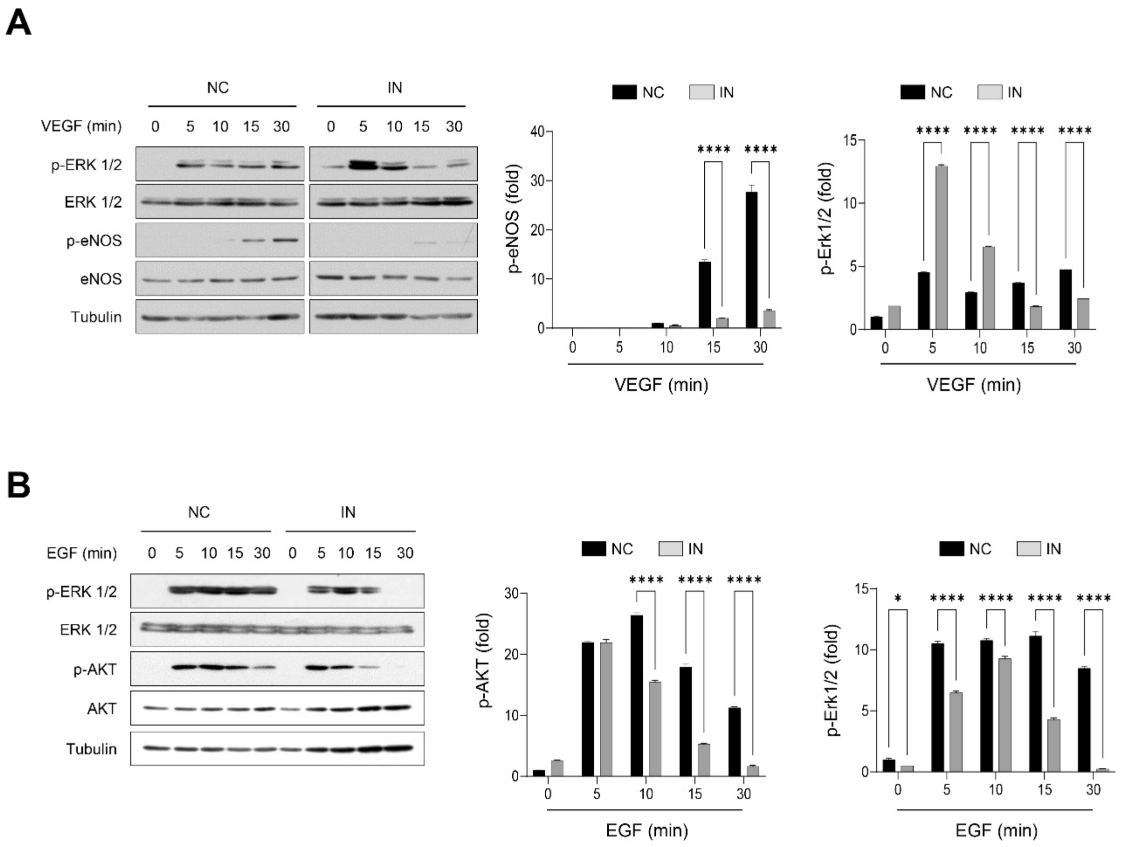

Supplementary Figure S7. PTPN12 is a potential target of miR-494-3p in HUVECs and A549 cells. (**A**) HUVECs were transfected with control (NC) or miR-494-3p inhibitor (IN), and were stimulated with VEGF (10 ng/ml) for the indicated periods of time. The expression levels of p-ERK1/2, p-ERK1/2, eNOS, and p-eNOS were determined by Western blotting. (**B**) A549 cells transfected with control (NC) or miR-494-3p inhibitor (IN), and were stimulated with EGF (10 ng/ml) for the indicated periods of time. The expression levels of p-ERK1/2, p-ERK1/2, AKT, and p-AKT were determined by Western blotting. The graphs represent densitometric analysis. n=3, ****P<0.0001.
