# Supplementary material for: Syntenin-1-mediated small extracellular vesicles promotes cell growth, migration, and angiogenesis by increasing onco-miRNAs secretion in lung cancer cells
Source: Cell Death Dis. 2022 Feb 8;13(2):122. doi: 10.1038/s41419-022-04594-2 (PMC8826407; doi:10.1038/s41419-022-04594-2)
Supplement: Supplementary file 9 — Supplementary Figure S8 [file 41419_2022_4594_MOESM9_ESM.pdf]

Supplementary Figure S8

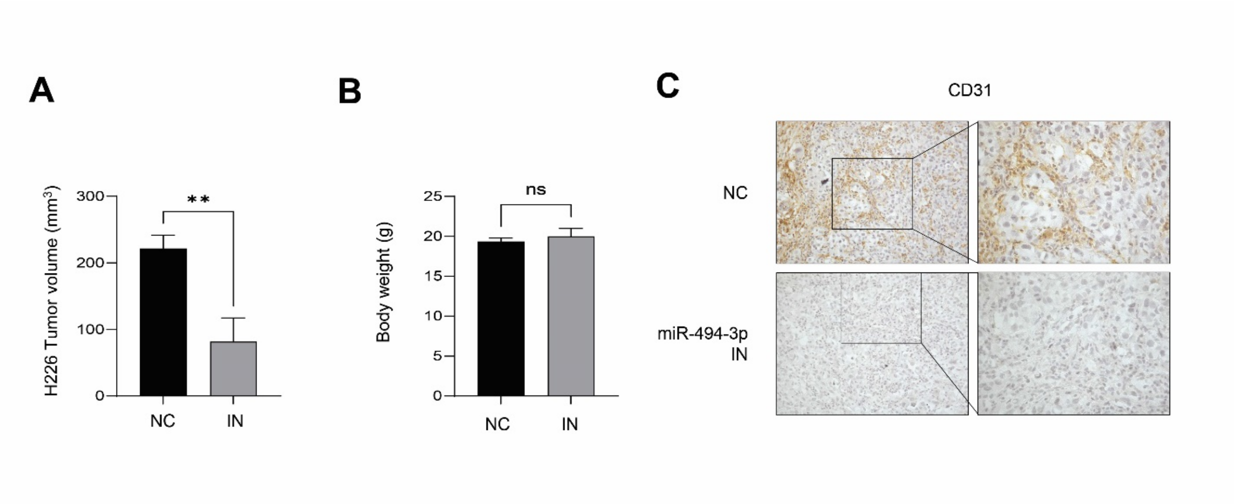

Supplementary Figure S8. miR-494-3p inhibitor suppresses tumor growth in NCI-H226 nude mice xenograft. (**A** and **B**) athymic Balb/c nude mice were subcutaneouly injected with NCI-H226 (2 $\times 10^6$ cells) into the right flank. When tumors reached a size of approximately 100 mm^3, negative control (NC) or miR-494-3p inhibitor (IN) was administered via intra-tumoral injection. Tumor volume (**A**) and body weight (**B**) at the end of experiment were shown (n = 6, **P<0.01). (**C**) Immunohistochemical staining for CD31 of NCI-H226 tumor sections treated with NC or miR-494-39 IN.
